# BanglaDial: A merged and imbalanced text dataset for Bengali regional dialect analysis

**DOI:** 10.1016/j.dib.2025.112200

**Published:** 2025-10-17

**Authors:** Mehraj Hossain Mahi, Anzir Rahman Khan, Mayen Uddin Mojumdar

**Affiliations:** Multidisciplinary Action Research Lab, Department of Computer Science and Engineering, Daffodil International University, Birulia, Dhaka 1216, Bangladesh

**Keywords:** Bangla language, Natural language processing, Bangla language identification, Dialects identification, Bangla regional dialects

## Abstract

BanglaDial is a unified corpus of Bengali dialectal text, consisting of 60,729 sentence-level entries representing eleven regional dialects and Standard Bangla. It reflects phonological, lexical, and syntactic divergences among dialects, offering a comprehensive linguistic spectrum beyond Standard Bangla. The dataset was compiled from online public repositories, representing regions such as Chittagong, Sylhet, Barisal, Rangpur, and others. All samples are written in Bengali script and annotated with their respective dialect labels, capturing key phonological, lexical, and syntactic variations across dialects. Bengali is the world’s seventh most spoken language, with over 265 million speakers, and is known for its vast literary heritage and linguistic complexity. However, existing NLP (Natural Language Processing) technologies are predominantly trained on Standard Bangla, leaving dialectal varieties significantly underrepresented and poorly supported in digital tools. BanglaDial addresses this gap by providing a curated and annotated resource to support automatic dialect identification and the development of dialect-aware natural language processing applications. This dataset also contributes to broader goals of linguistic preservation and digital inclusion for underrepresented Bengali-speaking communities.

Specifications Table

**Table:** 

Subject	Computer Sciences
Specific subject area	Underrepresented Languages, Bangla Dialects, Low-Resource Languages
Type of data	Table (text/string)
How data were acquired	The dataset is created by combining subsets of 4 corpora.
Data collection?	BanglaDial is created in two steps: 1) Data sources identification and 2) data preparation and insertion. Four publicly available corpora were identified, analyzed and filtered to build BanglaDial described in section data descriptions. Each corpus supports a set of multiple dialects.
Data source location	The list of the primary data sources used to create BanglaDial dataset is as follows. Primary data sources: Vashantor [4]: https://data.mendeley.com/datasets/bj5jgk878b/2 ONUBAD [5]: https://data.mendeley.com/datasets/6ft99kf89b/2 Bhashamul [6]: https://www.kaggle.com/competitions/regipa/data Bangla Dialect Dataset [7]: https://data.mendeley.com/datasets/sm63ryv5dt/1
Data accessibility	Repository name: Mendeley Data Data identification number: 10.17632/sx6ybcps2n.2 Direct URL to data: BanglaDial: A Merged and Imbalanced text Dataset for Bengali Regional dialect analysis. - Mendeley Data

## 1 Value of the Data

• BanglaDial is the first large-scale Bengali dialectal dataset that contains 11 regional dialects alongside Standard Bengali. It includes major areas of influence of dialects such as Chittagong, Sylhet, and Rangpur and Standard Bangla. Such a range makes it a significant sample of the collection of dialects of one of the world's most widely used languages.
• At present, many advanced generative AI models have limited exposure to low-resource languages which leaves Bengali dialects underrepresented. BanglaDial addresses this gap by serving as a foundational resource, enabling the development of translation systems, dialect identification frameworks, and multilingual chatbots capable of comprehending and producing text across these dialects. BanglaDial has practical applications in voice assistants, and educational apps that can adapt responses to a user’s dialect.
• Linguists and socio-linguists can use BanglaDial to perform corpus-based dialectometric, enabling research into phonological, lexical, and syntactic variations among regional dialects. The dataset is a useful tool for demographic analysis and geolocation in digital communication
• BanglaDial promotes diversity and inclusion in language technology by prioritizing the preservation and respect of Bengali dialects. It is also informative, with potential to provoke research and awareness about low-resource dialects in South Asia.
• The data exhibits imbalance due to the natural distribution of dialectal samples collected from various sources. This renders BanglaDial highly valuable for research in imbalanced data learning, e.g., research on resampling algorithms, cost-sensitive learning, data augmentation, and fairness-aware modelling for NLP.

## 2 Background

Bengali or Bangla is the seventh most spoken language in the world with close to 272.7 million native speakers in Bangladesh, parts of India, and a broad diaspora [1]. Although Bangla is widely used across formal, academic, and official contexts, natural language processing efforts have largely concentrated on Standard Bangla, with limited attention to the rich variation present in regional dialects.

In other regions of Bangladesh, people speak significantly different Bengali dialects, such as Chittagonian, Sylheti, and Barisali. The dialects often differ in pronunciation, grammar, and vocabulary, hence being difficult for Standard Bangla speakers to understand. Around 13 million people speak the Chittagonian dialect, 11.8 million speak Sylheti, and around 549,031 people speak the Barisali dialect in southern Bangladesh. Besides this, the diaspora Sylheti community in the UK includes around 400,000 speakers [2]. Such dialectic difference is a major impediment to communication and translation, particularly in online platforms, educational content, and public services. Therefore, the development of translation models, dialect identification systems, and language tools capable of processing regional Bangla dialects becomes increasingly important [3].

To meet these needs, the BanglaDial dataset was built. It is a merged and cleaned corpus derived from four public datasets of dialectal text samples in Bangla script: Vashantor [4], ONUBAD [5], Bhashamul (REGIPA) [6], and the Bangla Dialect Dataset [7]. Collectively, all these datasets cover twelve dialect regions in Bangladesh. Merging was done by identifying dialect-labelled corpora, cleaning texts, and placing them into a uniform format.

BanglaDial offers a vital resource to build inclusive NLP systems, improve dialectal translation acc, and enrich understanding of dialectal variation. Furthermore, because it is imbalanced in terms of its class distribution, it is also well-suited to research on data augmentation, resampling, and cost-sensitive learning that are vital for real-world language modelling.

## 3 Data Description

The objective of BanglaDial is to build a diverse and comprehensive dataset that captures a wide range of Bengali regional dialects, thereby supporting better generalization for dialect classification models. Our dataset design and collection methodology were inspired by the IADD dataset [8], which presents a large-scale corpus for Arabic dialect identification across multiple regions. Similarly, BanglaDial aims to capture dialectal diversity across Bengali-speaking regions in Bangladesh. BanglaDial is created in two main steps:

1. Data sources identification, and
2. Data preparation and insertion.

At the end of the process, BanglaDial is stored in a CSV format with the following key fields:

• **Sentence**: Contains the dialectal text sample written in Bengali script.
• **Language**: Indicates the corresponding dialect label used for classification.

**Fig. 1:**
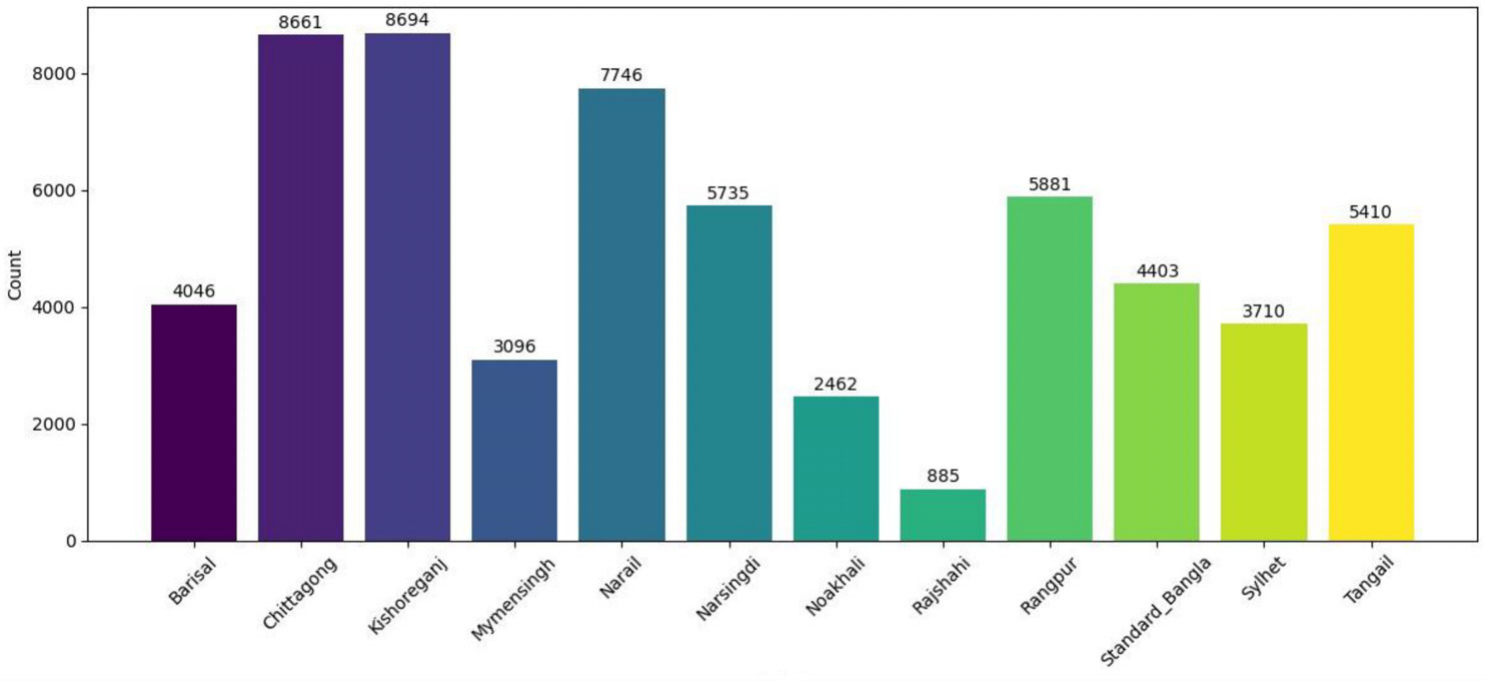
Dataset class wise description.

We have made our datasets openly accessible on a data repository [9]. There are 60,729 sentences in the dataset class wise distribution in Fig. 1.

**Fig. 2:**
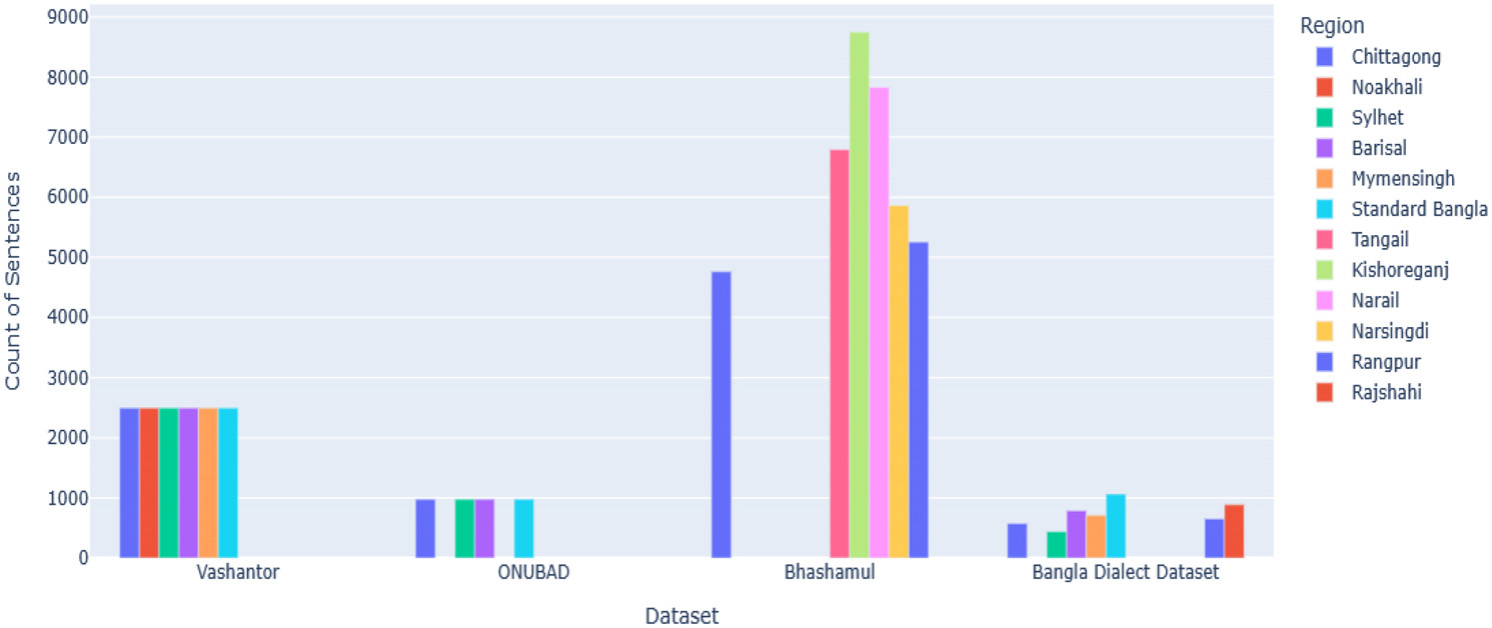
Sample size by region and by data source.

Table 1 presents a detailed breakdown of sentence counts and their corresponding percentages for each dialect. Additionally, Fig. 2 visualizes the origin of the dataset by mapping sentence contributions based on data sources. To further enrich the linguistic analysis, Table 2 shows the average word count, total word count, and average character count for each dialect, offering insights into the structural variations and verbosity across regional speech patterns within the dataset.

**Table 1:** Detailed overview of BanglaDial.

SL	Language	Sentences	Percentage
1	Chittagong	8661	14.26 %
2	Kishoreganj	8694	14.31 %
3	Narail	7746	12.76 %
4	Tangail	5410	8.90 %
5	Rangpur	5881	9.68 %
6	Narsingdi	5735	9.44 %
7	Standard_Bangla	4403	7.25 %
8	Barisal	4046	6.66 %
9	Sylhet	3710	6.11 %
10	Mymensingh	3096	5.09 %
11	Noakhali	2462	4.05 %
12	Rajshahi	885	1.46 %
	**Total**	60,729	**100%**

**Table 2:** Data description.

Region	Word Count	Avg Word Count	Avg Char
Chittagong	60,045	6.932802	36.148135
Kishoreganj	43,832	5.041638	25.629285
Narail	45,111	5.823780	28.756907
Tangail	31,557	5.833087	29.679482
Rangpur	35,391	6.017854	29.746642
Narsingdi	34,531	6.021099	30.950131
Standard Bangla	25,348	5.756984	29.849421
Barisal	23,476	5.802274	31.632229
Sylhet	22,361	6.027224	31.009973
Mymensingh	19,644	6.344961	33.809432
Noakhali	16,387	6.655971	34.500406
Rajshahi	4747	5.363842	29.526554
Total	362,430		

Table 3 provides a representative sample of the BanglaDial dataset, showcasing example sentences from each of the 12 regional dialects alongside their corresponding labels. The dataset is formatted as a CSV file, where each entry consists of two columns: the sentence and its associated dialectal class. This structure allows for straightforward parsing and label-based processing in natural language tasks. To illustrate phonetic and lexical differences, we have added transliteration, the standard Bengali and English equivalent for each example. The table demonstrates the phonetic, lexical, and syntactic variations that distinguish one dialect from another. The comparison with related dataset is given in Table 4.

**Table 3:** Example of the dataset.

Sentence	Transliteration	Standard Bangla	English	Dialects
আঁই যানি এডে বউত মাইফোয়া কমেন্ট ফরিবার লাই আইবো	Ãi jani ede bouth maifoẏa koment foribar lai aibo	আমি জানি এখানে অনেক ছেলে-মেয়ে মন্তব্য পড়তে আসবে	I know many boys and girls will come here to read the comments.	**Chittagong**
এলাই তো আন্নের সব্নাশ কইরা দিছে।	Elai to anner sorbonash koira diche	এরাই তো আপনার সর্বনাশ করে দিয়েছে	They are the ones who have ruined you.	**Kishoreganj**
দিপু কাকা আমারে ধরিছে বিটা যাতি অবেনে কাইলকে।	Dipu kaka amare dhoriche bita jati abene kailke	দিপু কাকা আমাকে ধরেছেন, কাল সেখানে যেতে হবে।	Uncle Dipu caught me, I have to go there tomorrow.	**Narail**
বাই, আমনেগো এনু কি ইকটু ফুন চার্জ দ্যান যাবো?	Bai, amnego enu ki ektu pun charj dyan jabo?	ভাই, আপনাদের এখানে কি একটু ফোন চার্জ দেয়া যাবে?	Brother, can I get a little phone charge here from you?	**Tangail**
আর ‍ফিরুজ হইচে উয়্যার বাপের মতোন।	Ar Firoz hoiche uyar baper moton	আর ফিরোজ হয়েছে ওর বাবার মতো।	And Firoz has become like his father.	**Rangpur**
ঘরের ভিত্তে গিয়াও হেই হমানে চিল্লান শুরু করছে।	Ghorer vitte giyao hei homane chillan shuru korse	ঘরে ভিতরে গিয়েও সেইভাবে চিৎকার শুরু করেছে।	As soon as he went inside the room, he started shouting in that way.	**Narsingdi**
আজকে আমার মন ভালো নেই	Ajke amar mon bhalo nei	আজ আমার মন ভালো নেই।	Today I am not in a good mood.	**Standard Bangla**
আব্বা মোর লইজ্ঞা লাল জামা কিন্যা আনছে	Abba mor loiggya lal jama kinya anshe	বাবা আমার জন্য লাল জামা কিনে এনেছে।	Father bought me a red shirt.	**Barisal**
আমি আফনার লাগি কিতা করতাম ফারি	Ami afnar lagi kita kortam fari	আমি আপনার জন্য কী করতে পারি?	What can I do for you?	**Sylhet**
আপনে কি এই ব্যাফারে কিসু কইতারবাইন	Apne ki ei byafare kisu koitarbain	আপনি কি এই বিষয়ে কিছু বলতে পারবেন?	Can you say something about this matter?	**Mymensingh**
ফোন হালাই লেপটিনে গেলে মনে অয় লাজেন জরুরী ফাইল হালাই অফিসে বসি রইছি	Phone halai leptine gele mone oi lajen joruri file halai office boshi roichi	যখন আমি আমার ফোনটি রেখে টয়লেটে যাই, তখন মনে হয় যেন একটা জরুরি ফাইল রেখে অফিসে বসে আছি।	When I leave my phone and go to the toilet, it feels like I am sitting in the office leaving behind an urgent file.	**Noakhali**
আমার চা ভাল্লাগে, আমার বুনের আবার কফি ভাল্লাগে	Amar cha bhal lage, amar buner abar coffee bhal lage	আমার চা ভালো লাগে, আমার বোনের আবার কফি ভালো লাগে।	I like tea, but my sister likes coffee.	**Rajshahi**

**Table 4:** Comparison with previous datasets.

Corpus	Data Source Type	Sentence	Number of Dialects	Dialects Language
IADD [8]	Secondary	135,804	6	Arabic
IIT Bombay Eng–Hindi Corpus [10]	Secondary	1492,827	2	English, Hindi
Arabic Dialect Identification [11]	Secondary	∼2.9M	5	Arabic
Our Dataset BanglaDial [9]	Secondary	60,729	12	Bangla

**Fig. 3:**
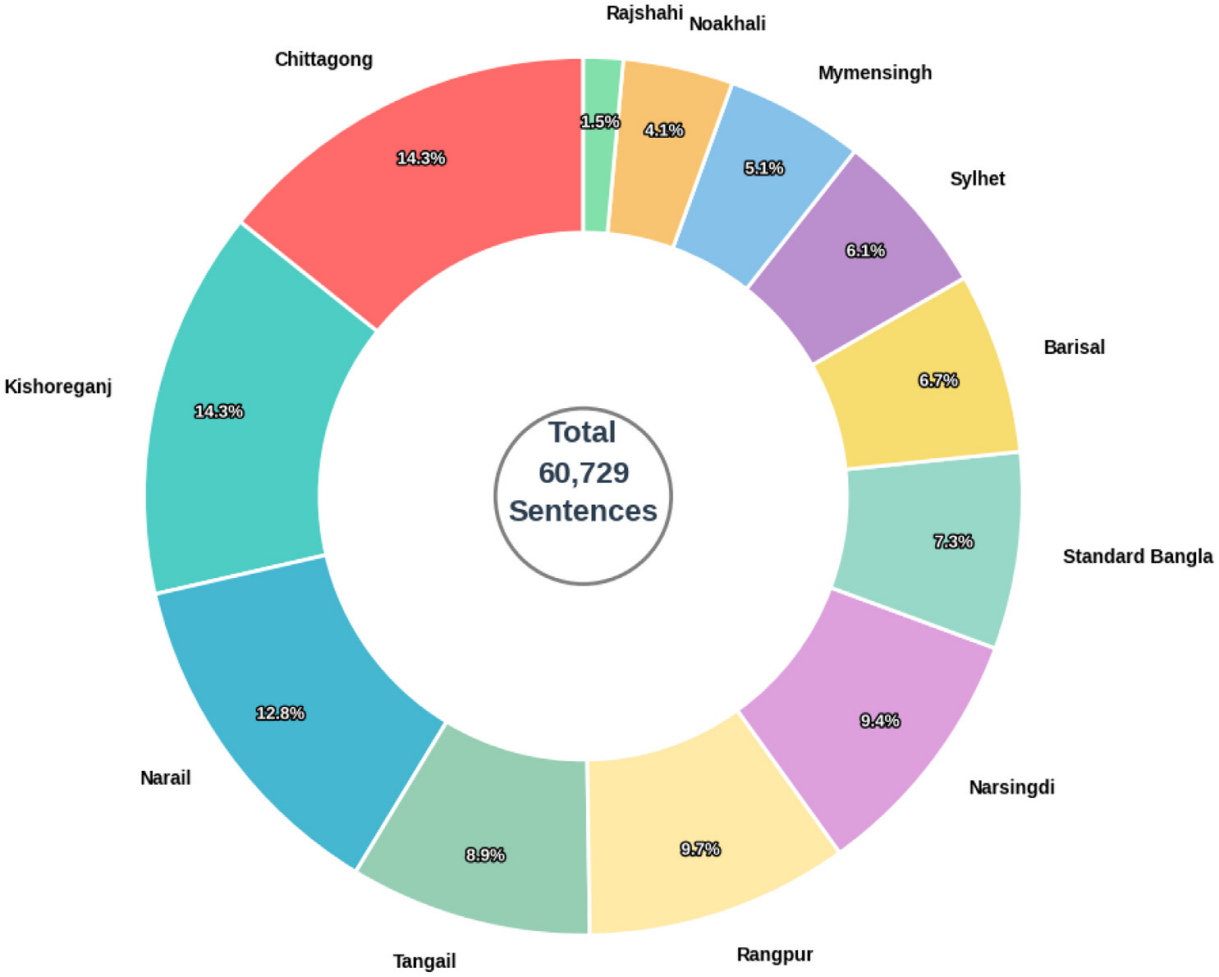
Proportional distribution class wise.

**Fig. 4:**
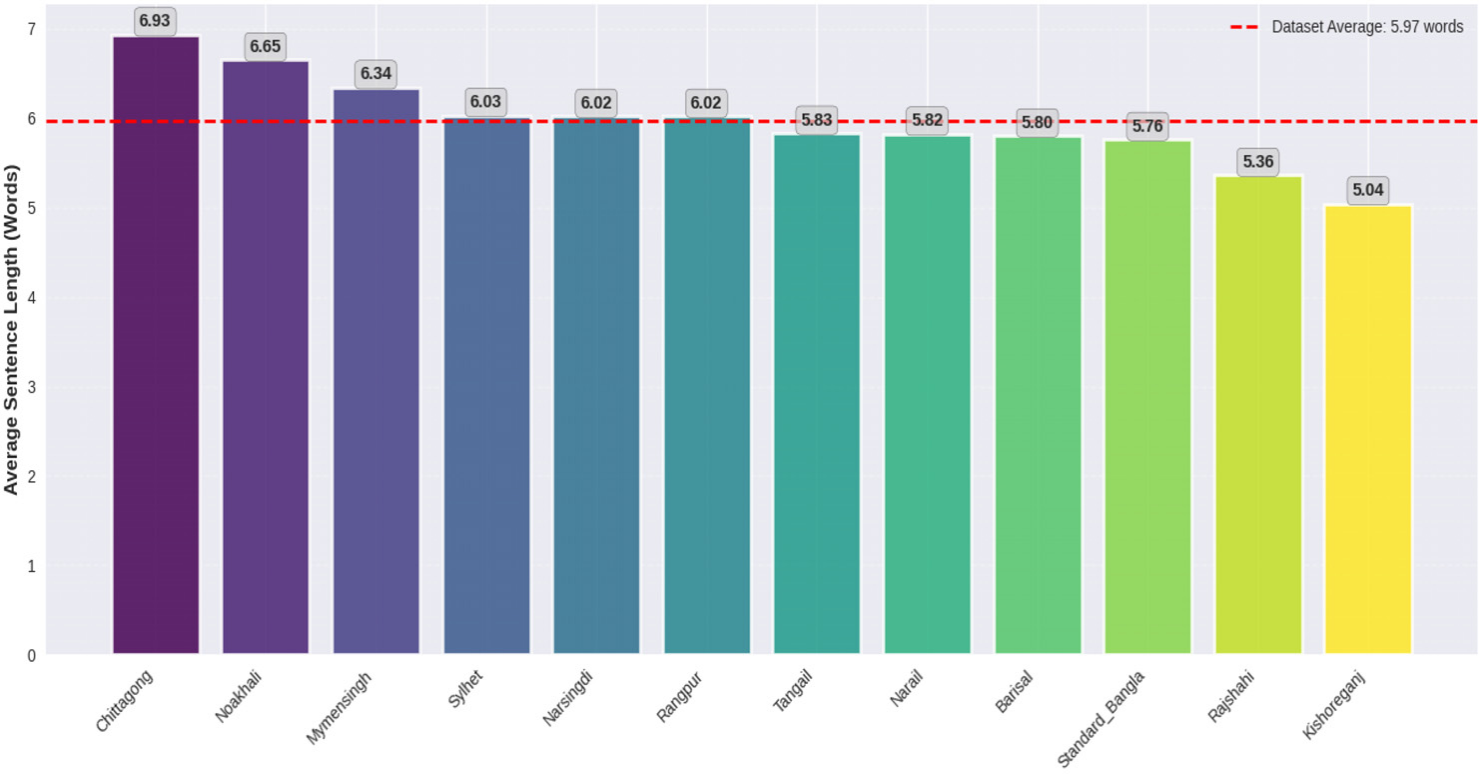
Average sentence length distribution by class.

Fig. 3, Fig. 4 present the dialect distribution and the average sentence length distribution respectively. Table 5 shows the overall imbalance indicators where class distribution exhibits moderate long-tail imbalance: the top-5 classes account for 60.5 % of all sentences (top-10 94.5 %); Shannon entropy [12] *H* = 3.416H=3.416H=3.416 (normalized 0.9530.9530.953); imbalance ratio IR [13]=9.82.

**Table 5:** Imbalance indicators.

Indicator	Value
Top-3 cumulative proportion	41.3 %
Top-5 cumulative proportion	60.5 %
Top-10 cumulative proportion	94.5 %
Shannon entropy (H, base-2)	3.416 bits
Normalized entropy H / log₂ C	0.953
Imbalance ratio (IR = max/min)	9.82

## 4 Experimental Design, Materials and Methods

### 4.1 Methodology

#### 4.1.1 Data sources identification

BanglaDial is constructed by combining subsets of four publicly available corpora: Vashantor, ONUBAD, Bhashamul, and the Bangla Dialect Dataset. The corpora consists of samples taken from varied collections of Bengali regional dialects, as listed in Table 6.

**Table 6:** Description of corpora composing our datasets.

Corpus	Source	Dialects (Regional Level)	Sentence	Number of Dialects
Vashantor [4]	Mendeley	Chittagong, Noakhali, Sylhet, Barisal, Mymensingh and Standard Bangla	15,000	6
ONUBAD [5]	Mendeley	Barisal, Sylhet, Chittagong and Standard Bangla	3920	4
Bhashamul [6]	Kaggle	Tangail, Kishoreganj, Narail, Narsingdi, Rangpur, and Chittagong	39,152	6
Bangla Dialect Dataset [7]	Mendeley	Rajshahi, Sylhet, Chittagong, Standard Bangla, Rangpur, Mymensingh, and Barisal	5131	7

The Vashantor corpus [4] includes dialectal Bengali sentences from various regions with a focus on lexical and phonological variation. ONUBAD [5] comprises a collection of translated texts of regional language usage in different socio-cultural contexts. Bhashamul [6], a competition dataset for dialect identification, comprises labeled data in a number of dialect zones focusing on classification tasks.Bangla Dialect Dataset [7] supplements dialectal differences collected from native Bangladeshis.

These resources were thoroughly reviewed, filtered, and combined to create a coherent yet imbalanced dataset, aimed at advancing research in dialect recognition, low-resource language processing, and computational sociolinguistics for Bengali.

#### 4.1.2 Data preparation and insertion

Data preparation and integration from individual sources into the BanglaDial dataset are outlined below.

#### 4.1.3 Vashantor

Sentences from the Vashantor corpus were sourced from Mendeley Data [4]. This dataset contains regional dialects from Chittagong, Noakhali, Sylhet, Barisal, Mymensingh, and Standard Bangla, totaling 6 dialect classes. The dataset is in CSV format and includes manually curated translations. Sentences were directly inserted into BanglaDial, with each instance labeled according to its regional tag. No major preprocessing was required beyond standard normalization and cleaning.

#### 4.1.4 ONUBAD

The ONUBAD dataset [5], also from Mendeley Data, provides translated dialectal sentences from Barisal, Sylhet, Chittagong, and Standard Bangla, spanning 4 dialect categories. Data entries are structured similarly to Vashantor. Sentences were directly added into BanglaDial, maintaining their original dialect labels. ONUBAD sentences required basic preprocessing, including the removal of punctuation and stop words, to align with BanglaDial’s formatting standards.

#### 4.1.5 Bhashamul

The Bhashamul dataset [6] was obtained from the REGIPA competition on Kaggle. It contains 6 dialect regions: Tangail, Kishoreganj, Narail, Narsingdi, Rangpur, and Chittagong. Sentences in this dataset were annotated at the utterance level and exported in CSV format. Only instances with clear dialect tags were retained. All entries were mapped to the appropriate region label before inclusion in BanglaDial. Minor inconsistencies in dialect spelling were normalized during preprocessing.

#### 4.1.6 Bangla dialect dataset

The Bangla Dialect Dataset [7] is the primary compilation resource for BanglaDial. It includes 7 dialects: Rajshahi, Sylhet, Chittagong, Standard Bangla, Rangpur, Mymensingh, and Barisal. This dataset integrates both crowd-sourced and institutional contributions and follows a structured CSV format with columns for sentence and dialect class. Sentences from this corpus were directly ingested into BanglaDial with minimal transformation, aside from character normalization and filtering of Latin script or noisy data.

#### 4.1.7 Data pre-processing

After collecting and merging the dataset, the dataset went through the following pre-processing and filtering stages-

1. Duplicate Removal

After merging the dataset, using pandas.drop_duplicates()all duplicate sentences were identified and removed. A total of 2574 duplicate sentences (≈ 4.1 % of all records). If the same sentence appeared more than once within or across files, only the first unique occurrence was kept.

2. Mentions and Hashtags Removal

Social-media style text often contains user mentions (e.g., @username) and hashtags (e.g., #topic). These were removed with regular expressions (@\*w*+ and #\*w*+).

3. Emoji Removal

Emoji symbols were identified through their Unicode ranges and removed from the sentences. While emojis often carry emotional meaning, they are not consistent linguistic markers of dialects, so they were excluded to keep the dataset text-focused.

4. Punctuation and Special Character Removal

Punctuation marks and other special symbols were removed, while retaining all Unicode letters, numbers. Sentences containing Latin-script contaminations were detected through Unicode-based regex filters.

5. Whitespace Normalization

Multiple spaces, unnecessary line breaks, and leading or trailing spaces were normalized sing regex (\*s*+).

6. Final Dataset

**Fig. 5:**
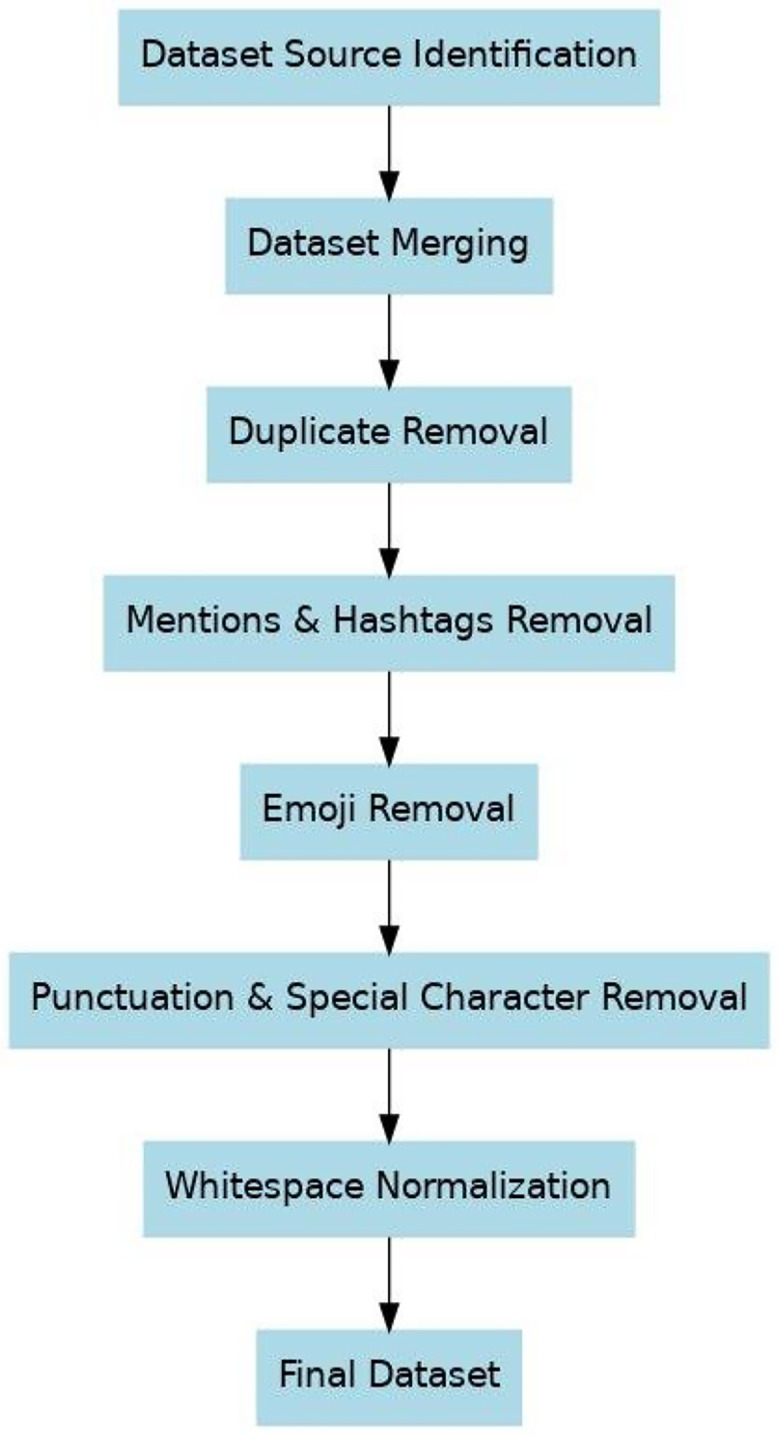
Flow diagram.

After all preprocessing steps, the dataset contained two standardized columns: sentence (cleaned Bangla text) and label (the original region name derived from the dataset file). The final dataset was saved in both CSV and XLSX formats. After cleaning and filtering dataset the sentences remained from 63,303 to 60,729. The overall workflow is illustrated in Fig. 5. All related tables, figures, and supplementary materials are available at: https://github.com/Mehraj-Hossain-Mahi/BanglaDial.

## Limitations

BanglaDial is a valuable resource for Bengali dialectal analysis, there are certain limitations:

• Unbalanced Distribution: The corpus reflects natural imbalances in dialect usage, with certain dialects of regions (e.g., Standard Bangla, Chittagonian) being more represented than others. This can bias machine learning algorithms unless balancing methods are employed in addition.
• Text-Only Representation: All the samples are in text form in Bengali script. The absence of phonetic or audio data prevents detailed phonological and prosodic analysis, which is crucial for comprehensive dialect studies.
• Source Variability: Because the data were collected and concatenated from different online sources and corpora, inconsistency in annotation style, dialect designation, or linguistic formatting may be present.
• No Contextual Metadata: There is no gender, socio-economic status, or age speaker metadata available in the dataset, which would be important in demographic or sociolinguistic research.

These constraints present an opportunity for further studies to enlarge, improve, and expand the dataset for additional usefulness in low-resource NLP and dialectology.

## Ethics Statement

The authors declare that there are no ethical concerns regarding the creation of the BanglaDial dataset. All data were obtained from publicly available online sources and existing open-access corpora, in full compliance with their respective usage and redistribution policies. The dataset is fully anonymized and does not contain any personally identifiable information. No direct human subjects were involved in the data collection process, and the data used were originally collected under conditions that ensured voluntary and informed contributions.

## Credit Author Statement

**Mehraj Hossain Mahi**: Methodology, Visualization, Writing–original draft; **Anzir Rahman Khan**: Conceptualization, Validation, Writing–review & editing; **Mayen Uddin Mojumdar**: Validation, Supervision

## Declaration of generative AI and AI-assisted technologies in the writing process

During the preparation of this manuscript, the authors used ChatGPT for the manuscript's language improvement. After using this tool/service, the authors reviewed and edited the content as needed and took full responsibility for the content of the publication.

## Data Availability

Mendeley DataBanglaDial: A Merged and Imbalanced text Dataset for Bengali Regional dialect analysis. (Reference data)
